# Anomalous Origin of the Left Main Artery from Right Coronary Sinus with a Prepulmonic Course

**DOI:** 10.5334/jbr-btr.872

**Published:** 2015-12-30

**Authors:** Hatice Kaplanoglu, Osman Beton, Lale Dinc, Hakki Kaya, Burhan Yilmaz

**Affiliations:** 1 Diskapi Yildirim Beyazit Research and Training Hospital, Ankara, TR; 2 Cumhuriyet University, Faculty of Medicine, Department of Cardiology, Heart Center Hospital, Sivas, TR

**Keywords:** Anomalous coronary artery, coronary vessels, chest pain, coronary CT angiography, diagnosis

## Abstract

A 32 year old female patient presented to the cardiology clinic with an atypical chest pain. Her history revealed no other condition than Leopard syndrome which was diagnosed on her birth. On her coronary CT angiography, LMCA originated from the right coronary sinus and had a prepulmonic course. The purpose of this article is to present this patient with Leopard syndrome accompanied by left coronary artery outlet and coronary sinus abnormality.

## Introduction

Congenital anomalies of the coronary arteries are incidentally detected during coronary angiography or autopsy. Such anomalies may result with life threatening conditions such as arrythmia, syncope, myocardial infarction and sudden death [1]. The most important finding affecting the prognosis is the course with respect to the aorta and the pulmonary artery. Abnormal outlet of the left main coronary artery (LMCA) from the left valsalva sinus is less common than the abnormal aoutlet of the right coronary artery (RCA) from the left valsalva sinus [2]. The coronary sinus is the largest venous structure of the heart. Congenital anomalies of the coronary sinus might be isolated or associated to congenital heart diseases [3]. Coronary sinus anomalies are very rare, and may present without cardiac symptoms or cardiac dysfunction [3]. Multidetector computed tomography (MDCT) is a non-invasive imaging method for comperhensive evaluation of cardiac malformations. Leopard syndrome is an extremely rare hereditary disorder characterized with abnormalities of the skin, heart structure and function, the inner ear, craniofacial region and/or genital region. Individuals with this disorder have variable range and severity of symptoms, and somatic features. The coronary CT angiography of a female patient with Leopard syndrome, obtained due to atypical chest pain showed valvular pulmonary stenosis, left coronary artery anomaly originating from the right with prepulmonic course. The literature search showed that this patient was the first case with Leopard syndrome accompanied by left coronary artery outlet and coronary sinus abnormality.

## Case

**Figure 1:**
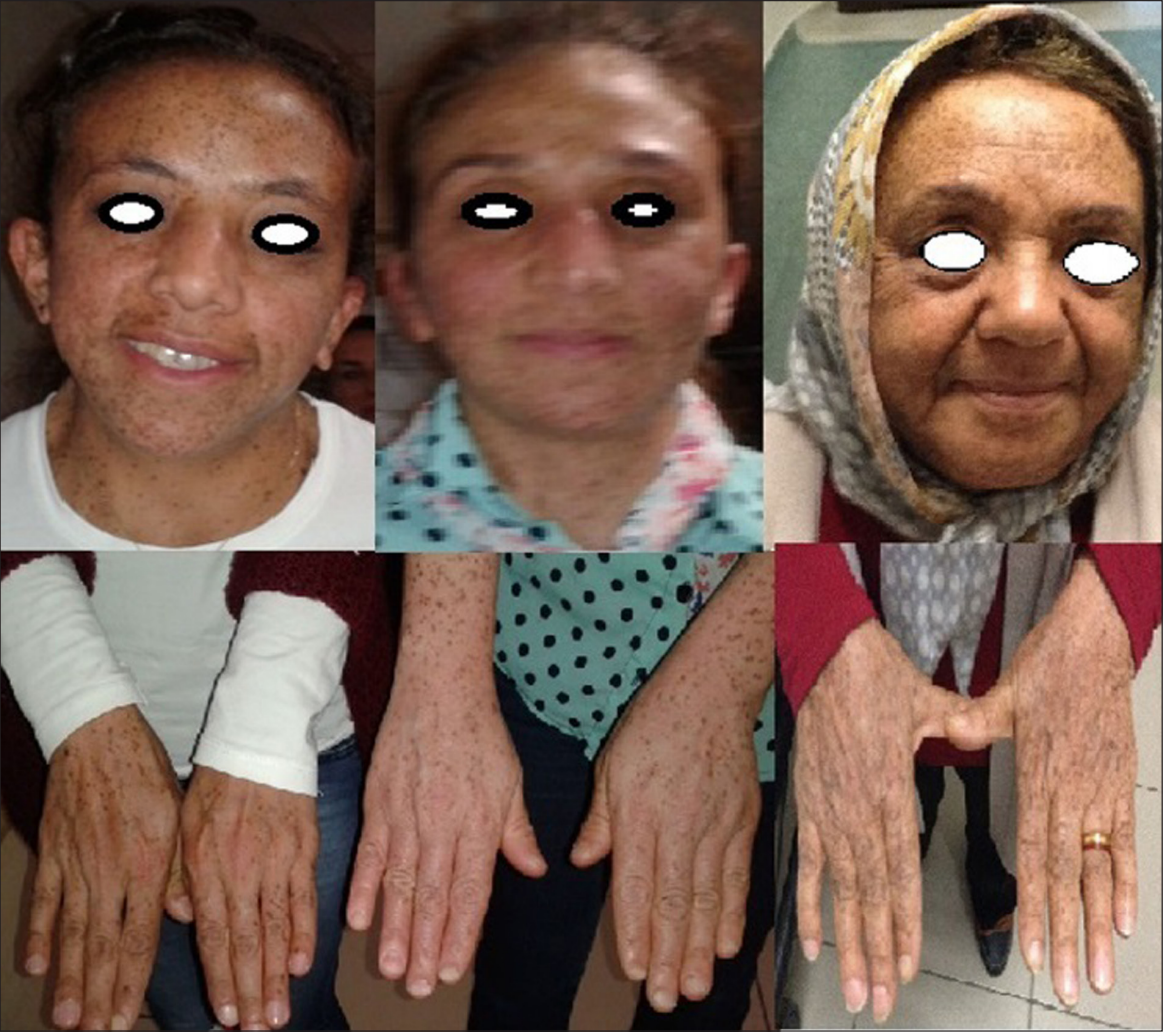
Our patient (on the left), her older sister (in the middle) and their mother (on the right) with multiple lentiginous lesion on their faces and hands.

**Figure 2:**
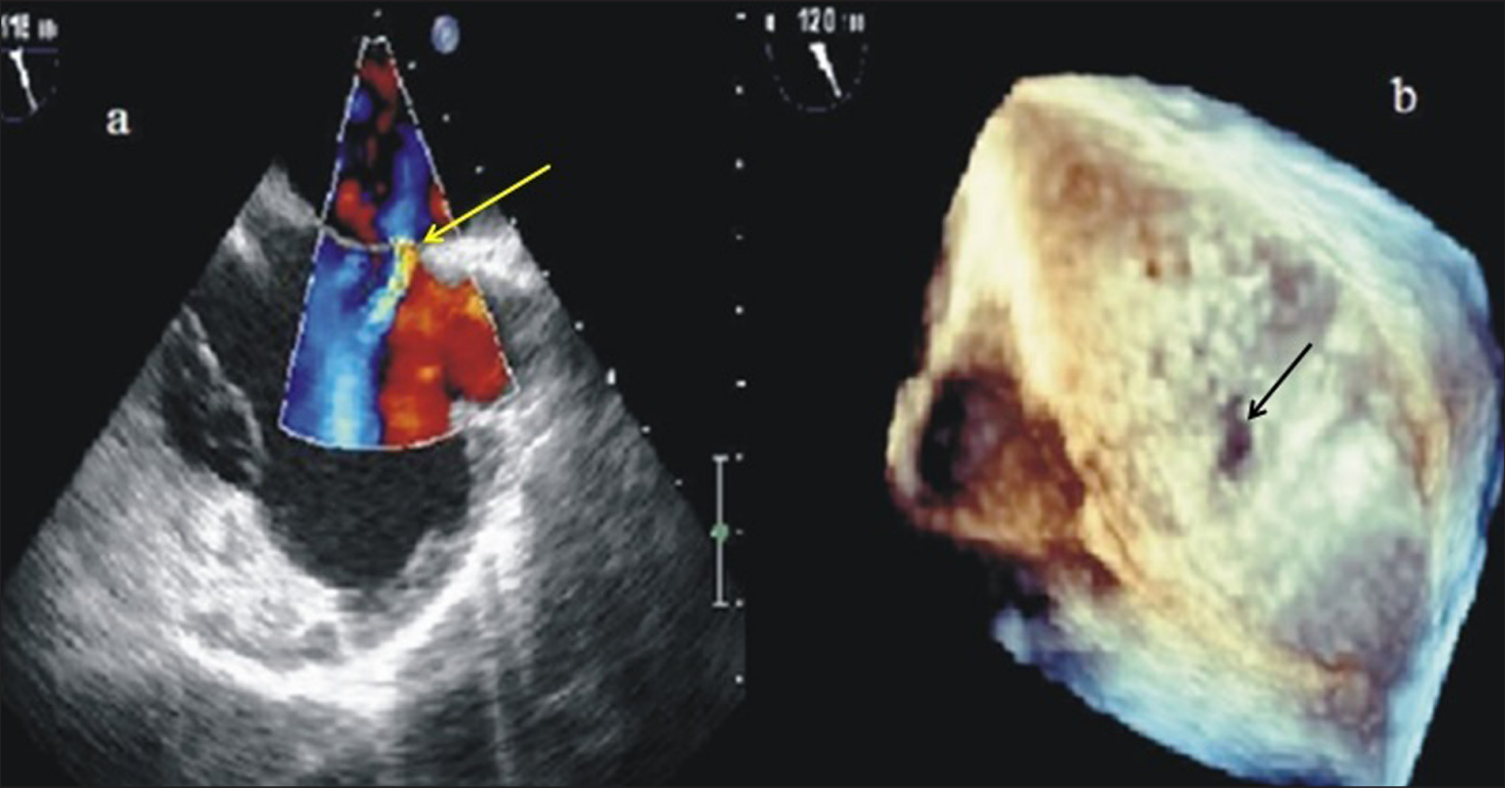
Echocardiography showing atrial septal defect image on color Doppler imaging on bicaval position (a) (yellow arrow) and on 3D imaging (b) (black arrow).

A 32 year old female patient presented to the cardiology clinic with an atypical chest pain. Her history revealed no other condition than Leopard syndrome which was diagnosed on her birth. However, her family history revealed that her older sister who also had Leopard syndrome, was diagnosed with pulmonary stenosis 5 years ago and underwent successful baloon valvuloplasty. The patients mother, who also had Leopard syndrome had undergone open heart surgery due to pulmonary stenosis 20 years ago and 5 years earlier she had had percutaneous coronary artery angioplasty due to coronary artery disease. On her physical examination; pectus excavatum, multiple lentiginous lesions on the face and hands (Figure 1), ocular hypertelorism, and short height were detected. Sinus rythm and left branch block was detected on her electrocardiography. Her echocardiography was as follows: left ventricular ejection fraction 60%, normal heart chambers, atrial septal defect (4mm) (Figure 2a, 2b), low grade pulmonary valve stenosis (maximal gradient 18 mmHg, mean gradient 9 mmHg), right ventricular pressure 20 mmHg.

**Figure 3:**
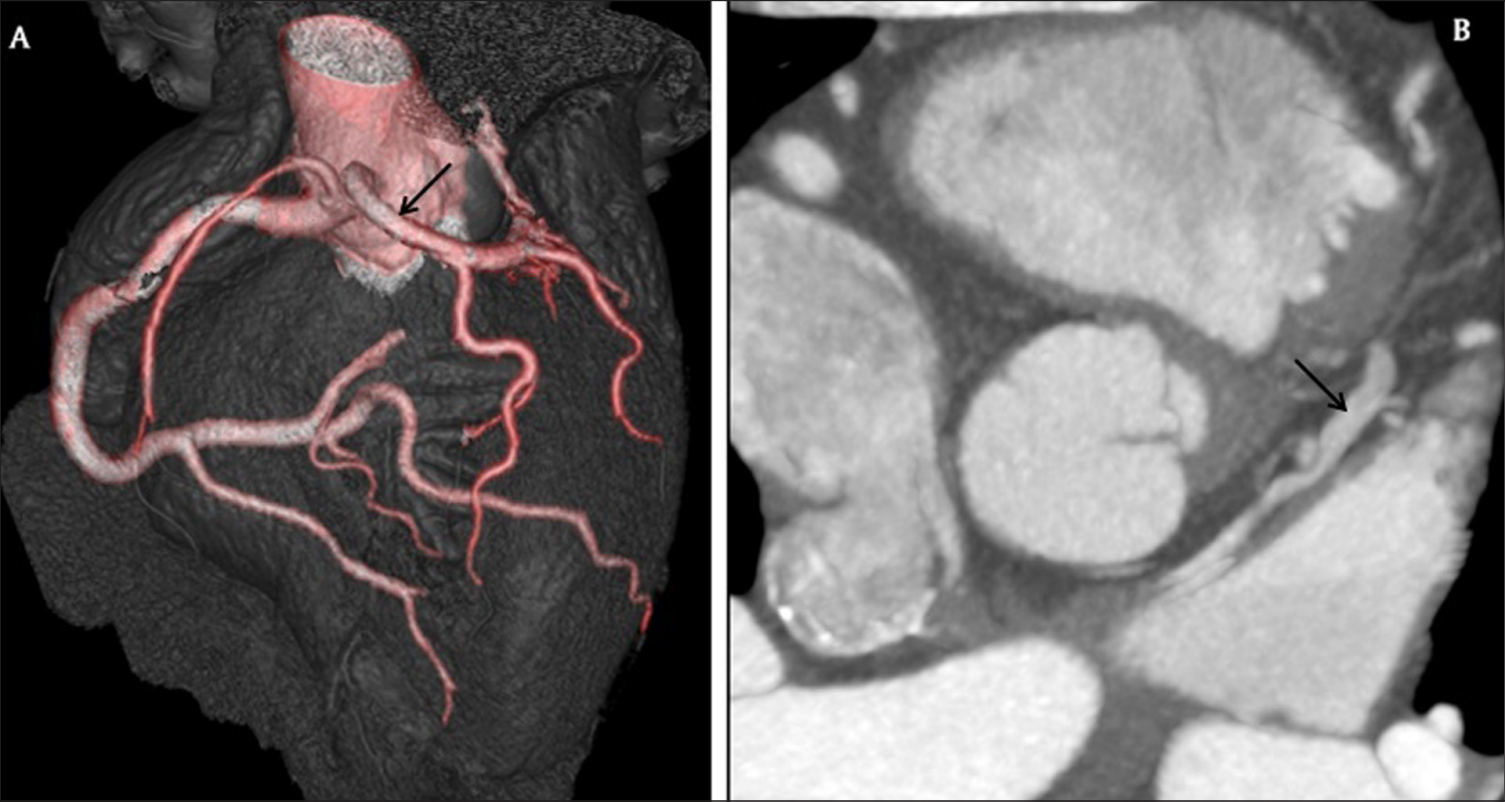
3a: Coronary CT angiography showing the origin of left main coronary artery (black arrow) from the right coronary sinus with prepulmonic course; 3b:Coronary CT angiography showing the anterior interventricular vein (black arrow) draining directly to the left atrium.

On her coronary CT angiography, atrial septal defect, 3 mm in its widest place, widened left pulmonary artery (diameter 34 mm), mild thickening of the pulmonary semilunar valves were detected. The right coronary artery was dominant and diffusely ectatic (diameter: 7.5 mm widest). The right ventricular branch of the right coronary artery was directly originating from the sinus of Valsalva and this branch also gave the conus branch. PDA and PLB reached the apex through the interventricular sulcus. LMCA originated from the right coronary sinus and had a prepulmonic course (Fig. 3a, 3b). LMCA (6,5 mm) and LAD (6,2 mm) were ectatic throughout their course. Circumflex artery structure and callibration were normal. The structures of the large cardiac vein and coronary sinus were normal. The anterior interventricular vein was draining directly to the left atrium.

Coronary angiography revealed similar findings as the coronary CT angiography. Maximum gradient of 20 mmHg was observed in pulmonary valve hemodynamic study. As a result of the investigations it was decided to follow up the patient with medical treatment.

## Discussion

Coronary artery anomalies are very rare, and are seen in less than 1% of the general population [4]. Particularly, LMCA originating from the right sinus of Valsalva (RSV) is extremely rare, and it is incidentally found in approximately 0,017% of all coronary artery angiographies [5].

The most important factor affecting long-term prognosis is the course of left coronary artery and its position regarding the aorta and the pulmonary artery. Left coronary artery originating from the right sinus of Valsalva might have 4 courses: between the aortic root and the pulmonary artery (interarterial course), transseptal course (subpulmonic course), anterior course originating from the right ventricle (anterior or prepulmonic course), posterior course regarding the aortic root (retroaortic course). Left coronary artery lying between the aorta and the pulmonary artery has the highest rate of sudden death among young males [6]. In some cases, abnormal coronary artery with interarterial course may result in ischemia or sudden cardiac death [7].

There are different hypotheses regarding poor prognosis due to abnormal coronary arteries originating from the right sinus of Valsalva. The first one is that exercise induced pressure between the pulmonary trunk and the aorta may lead to decreased coronary blood flow [8]. The second implies that, acute takeoff or hiatus like orifice in these arteries may lead to ischemia resulting in angina, syncope, congestive heart failure, arrhytmia and/or sudden death [5]. The third, being myocardial remodelling is related to poor prognosis due regional ischemic inflammatory histopathological changes [10]. And lastly, some authors advocate the hypothesis that coronary arteries with abnormal course have tendency of atherosclerosis [4]. In the presented study the risk of ischemia and sudden death is low.

Coronary sinus is the largest venous structure. Coronary sinus anomalies may be isolated or related with congenital heart diseases [3]. Some coronary sinus anomalies may change the cardiac hemodynamics, resulting in clinical signs and symptoms and requiring definite diagnosis and treatment. However, some anomalies may present without any clinical symptoms [3]. In the presented case, the coronary anomaly was accompanied with ASD and an abnormal venous structure draining in the left atrium. No such case to our knowledge has been reported in the literature.

## Competing Interests

The authors declare that they have no competing interests.
